# Melanoma: Staging and Follow-Up

**DOI:** 10.5826/dpc.11S1a162S

**Published:** 2021-07-01

**Authors:** Chryssoula Papageorgiou, Zoe Apalla, Sofia-Magdalini Manoli, Konstantinos Lallas, Efstratios Vakirlis, Aimilios Lallas

**Affiliations:** 1 Second Dermatology Department, Medical School, Faculty of Health Sciences, Aristotle University of Thessaloniki, Greece; 2 First Dermatology Department, Medical School, Faculty of Health Sciences, Aristotle University of Thessaloniki, Greece

**Keywords:** Melanoma, staging, follow-up

## Abstract

Cancer staging is the process determining to which extent a cancer has spread and where it is located in the body. A thorough staging is of utmost importance, not only because it provides the most accurate prognostic estimation, but also because several crucial decisions, such as the treatment choice and the follow-up strategy, vary according to the tumor’s stage. The current staging system for melanoma is based on the 8^th^ edition of TNM classification issued by the American Joint Committee on Cancer (AJCC) in 2017. It includes a clinical and a pathological staging, both consisting of 5 stages (0–IV). The stage of a melanoma is determined by several factors, among which the Breslow thickness, the pathological presence or absence of ulceration in the primary tumor, the presence and the number of tumor-involved regional lymph nodes, the presence or absence of in-transit, satellite and/or microsatellite metastases, and the presence of distant metastases. Following melanoma diagnosis, an accurate medical workup, in line with the stage and the physical examination, should be performed. A continuous patient monitoring is fundamental to detect a potential relapse or a second primary melanoma and should be lifelong. However, there is still no universally adopted follow-up strategy program and different follow-up schemes have been suggested. Future prospective studies are needed to evaluate different follow-up protocols according to the adopted therapy, as novel recent therapies (targeted and immunotherapies) are being increasingly used.

**Key Messages**
- Proper staging is of utmost importance because it provides accurate prognostic estimation. Several crucial decisions, such as the treatment choice and the follow up strategy, are based on the tumor stage.
- Physical examination during staging procedure and follow-up visits are important to avoid unnecessary imaging and laboratory tests that could increase the patients’ anxiety. A personalized approach taking into consideration the patient’s risk factors, is strongly recommended.
- Melanoma patients should be kept under surveillance lifelong due to an increased risk of developing a second primary melanoma and the risk of recurrence. Higher intensity follow-up strategies during the first 5 years are recommended due to higher rates of regional or distant relapse.

## Introduction

Staging is a process determining the extent to which a cancer has spread in a person’s body and where it is located. Cancer stage is categorized from 0 to IV, with stage IV cancer corresponding to a cancer that has metastasized at distant locations. The most used system to stage solid tumors, including melanoma, is the universally accepted TNM (Tumor, Node, Metastasis) staging system. Cancer staging can be divided into clinical and pathological staging. Clinical and pathological stages are defined by different criteria and may differ but are generally considered as complementary to each other. In general, clinical staging is based on all the available information obtained before surgical excision of the tumor (eg by physical examination, blood tests, and imaging), while pathological staging is performed by a pathologist and relies on the information provided by microscopic examination of the tumor following surgical resection.

The clinical stage of a melanoma can be determined only following a complete excision of the primary tumor, a clinical examination of the skin and lymph nodes, and a radiologic assessment for regional and distant metastases’ detection. Pathological staging of a melanoma takes into account not only the microstaging of the primary tumor and the wide excision but also considers the information on regional lymph nodes after partial or complete lymphadenectomy, when performed. A proper staging is extremely important, because it provides the most accurate prognostic estimation and allows to take several crucial decisions, such as the treatment choice and the follow-up strategy, that are based on clinical tumor stage.

### Melanoma Staging System

The current staging system is based on the 8^th^ edition of TNM classification for staging of melanoma issued by the AJCC in 2017 and is summarized in Tables 1–5 [1]. This relatively new system has been broadly accepted after its publication and is considered the cornerstone for classifying melanomas [2,3].

There is both a clinical and a pathological staging, both consisting of 5 stages as follows:

Clinical Staging:

- 0: in situ disease
- I and II: localized disease

Stage I is further divided into substage IA and IB, while stage II includes substages IIA, IIB and IIC. The determining factors for staging and substaging are the Breslow thickness and the presence or absence of ulceration after the pathological assessment of the primary tumor (Tables 1 and 2). Of note, mitotic rate and Clark’s level of invasion, previously used for sub-classification, no longer influence melanoma staging.

- III: regional disease

Regional disease is defined by the presence of metastases in regional lymph nodes and/or “in transit metastases”, “satellite metastases”, and microsatellite metastases. Satellite metastases are defined as cutaneous or subcutaneous metastatic lesions up to 2 cm from the margin of the primary tumor. In-transit metastases are defined as cutaneous or subcutaneous lesions located between 2 cm from the primary tumor and the regional nodal basin. Microsatellite metastases are defined as tumor nests larger than 0.05 mm in diameter in the reticular dermis, subcutis, or vessels beneath the primary invasive tumor, but separated from it by at least 0.3 mm of normal tissue on the section in which the Breslow measurement was taken.

Regional lymph nodes metastases are defined as metastases in the lymph node basin that drains lymph from the region around the tumor. Involvement of regional lymph nodes is confirmed by their pathological examination after sentinel lymph node (SLN) biopsy (for clinically occult lymph node metastases) or therapeutic lymph node dissection when performed (for clinically evident regional lymph node disease). Involvement of regional lymph nodes may be also detected by clinical, radiologic examination and/or diagnostic biopsies (clinical staging). Therefore, there is only 1 stage group for clinical stage III. In contrast, pathological stage III is divided into A, B, C, and D stage groups depending on Breslow thickness, the pathological presence or absence of ulceration in the primary tumor, the number of tumor-involved regional lymph nodes, and the presence or absence of in-transit, satellite and/or microsatellite metastases (Table 4).

- IV: distant metastatic disease

This stage includes distant metastases to lung, central nervous system (CNS) or other organs as well as to skin, soft tissue and nonregional lymph nodes. Although there is no further division to substages, a sub-classification according to the number of organs involved, which organs are involved, and serum levels of lactate dehydrogenase (LDH) is essential for prognostic reasons (Table 5).

### Staging workup

#### Histopathologic Examination

When a suspicious lesion is detected, a biopsy should be performed. A narrow-margin (1–3 mm) excisional biopsy is strongly preferred. In case of primary melanoma, the histopathological features along with clinical examination are determining factors for staging and further management. Therefore, the pathology report should include the Breslow thickness, the ulceration status, the dermal mitotic rate, the margin status, the presence, or absence of microsatellitosis, and the presence or not of pure desmoplasia.

#### Physical Examination

Special attention should be paid to the physical examination of the entire skin surface to look for satellites or in-transit metastases but also for a second primary melanoma. Physical examination of the regional lymph node basin should be included.

#### Sentinel Lymph Node Biopsy and Imaging

Patients with a melanoma in situ and a clinical stage IA melanoma with normal physical examination and no other symptoms need no further imaging or laboratory tests. They also are not candidates for SLN biopsy at baseline. The staging procedure is completed with the performance of wide excision [1].

Patients with clinical stage IB melanoma with normal physical examination and no other symptoms need no further imaging or laboratory tests at baseline. Concerning SLN biopsy, this should be considered in patients with T1b melanoma. The decision depends on several factors, such as comorbidities, age, mitotic rate or lymphovascular invasion [1]. Patients with a T2a melanoma, should undergo SLN biopsy.

Patients with clinical stage II melanoma with normal physical examination and no other symptoms need no further imaging or lab tests at baseline, but a SLN biopsy should be offered [1,4,5].

In melanoma patients of any stage, if an equivocal regional lymph node is detected during clinical examination, an ultrasound (US) should be considered prior to SLN biopsy. However, a negative nodal basin US is not a substitute for biopsy of clinically suspicious lymph nodes and histopathology should be warranted. Moreover, abnormalities or suspicious lesions on nodal basin US should be histopathologically confirmed. The presence of lymph node metastasis can be confirmed either with core biopsy or fine-needle aspiration (FNA) [6–8]. Similarly, if clinical or microscopic satellite/in-transit metastases are suspected, a biopsy is mandatory.

If a SLN biopsy is indicated, it should be performed at the same time with the wide excision of the primary melanoma. Noteworthy, SLN biopsy was shown to have only prognostic (and not therapeutic) significance [9–13]. A positive SLN biopsy would directly upstage a patient to stage III, which highlights its significance as a staging procedure, especially after the introduction of adjuvant systemic therapy for stage III. A complete lymph node dissection is not anymore recommended in case of positive SLN biopsy, since it does not offer any therapeutic benefit, it has little prognostic value, and is associated with surgical morbidity [14–17]. It is, however, indicated for the treatment of lymph node metastases diagnosed clinically or by imaging, in the absence of distant metastases.

Imaging for baseline staging should be considered in patients with pathological stage IIIA melanoma and should be performed in all patients with stage IIIB/C/D [1]. Imaging modalities include chest/abdominal/pelvic CT with intravenous (iv) contrast or whole-body PET/CT, with or without brain MRI with iv contrast. Moreover, if clinically indicated, neck region should be also checked with CT with iv contrast.

Finally, stage IV melanoma patients need careful total body medical imaging (CT or PET/CT, brain MRI). Moreover, plasma LDH should also be assessed [1].

### Follow-Up

After melanoma diagnosis, the role of ongoing surveillance of disease-free patients is of paramount importance. The main goals of the follow-up are the following:

1. Early identification of relapse (local, distant) and subsequent guidance for adjuvant treatment, where appropriate.
2. Early detection of a second primary melanoma and/or non-melanoma skin cancer.
3. Recognition and management of side-effects, in case of adjuvant systemic treatment.

Early detection of relapse is associated with a higher survival rate, highlighting the importance of an adequate follow-up. The likelihood of recurrence varies according to melanoma stage at first presentation. Patients with melanoma in situ, are very unlikely to recur following wide excision. There are a few exceptions though, such as lentigo maligna type [18–20]. In general, patients with earlier stage melanoma at first presentation are less likely to recur compared to those with more advanced stages. Accordingly, the timing of relapse varies according to the stage. Patients with advanced melanoma tend to recur more quickly compared to those with earlier stage [21–23]. Nonetheless, the vast majority of relapses are recorded in the first 5 years and most of them within 2–3 years following surgery. Moreover, the risk of recurrence tends to decrease over time for melanoma stages, but late recurrence (more than 10 years after the initial diagnosis) cannot be excluded [21,22,24–26]

Patients with a personal history of melanoma are at high risk of developing a second primary melanoma. Concerning the risk of developing a second primary melanoma, data reported in the literature is very heterogenous. The reported percentage of melanoma patients developing a second primary melanoma ranges between 2% and 20% [23, 27–30]. In a cohort of prospectively monitored melanoma patients, the cumulative 5-year risk of second primary melanoma was 8% [30]. Interestingly, the risk appears to be higher within the first year after the diagnosis of the first melanoma, but it remains considerable for at least 5 years and very possibly even more [23, 27–30]. Therefore, individuals with melanoma history should rather be considered at a life-long increased risk of developing a new primary melanoma.

Although the need for a follow-up in patients with melanoma is not a matter of debate, surveillance recommendations vary widely in terms of methods and frequency of visits, and examinations. As there is currently lack of evidence regarding the efficacy of follow-up strategies, different follow-up schemes have been proposed and are mainly based on expert opinions. The suggested follow-up schemes consider the melanoma stage and the presence or not of additional risk factors.

As mentioned above, the first 5 years following the excision of the primary tumor are the most crucial due to high rates of relapse. This is why current guidelines suggest adopting higher intensity follow up strategies during this period. Still, because of the lifetime increased risk of a second primary melanoma or a non-melanoma skin cancer, as well as the risk for late recurrence, monitoring programs for melanoma patients should go beyond 5 years, including at least 1 strongly recommended annual skin exam lifelong [31].

The modalities used to monitor melanoma patients include whole body skin examination, physical examination of the regional lymph nodes, blood tests, and imaging exams, such as chest X-ray, ultrasound, CT, PET/CT, and MRI. More analytically, a clinical evaluation performed by a dermatologist is mandatory at any stage and includes a total body skin examination (with or without a total body clinical and dermoscopic digital documentation) to identify local recurrences (scar, satellite/in-transit recurrence) and subsequent primary melanoma or other skin cancers. Clinical evaluation should also include the examination of the regional lymph nodes and the evaluation of patients’ symptoms and/or signs that would direct appropriate imaging if needed. Ultrasound of the lymph nodes is the most accurate method to detect nodal disease and is generally recommended in patients with equivocal lymph node during physical examination, in patients with AJCC T1b stage and above, in patients who were offered SLN biopsy but it was not performed or in patients with positive SLN biopsy who did not undergo complete lymph node dissection [32]. Other imaging modalities (CT, PET/CT, MRI, chest x-ray) should be considered for monitoring asymptomatic patients in more advanced stages or when signs and symptoms may suggest distant metastasis [33]. In any clinical scenario, if there is a recurrence suspect, this should be confirmed by histopathologic analysis whenever possible.

Finally, routine blood testing (LDH, S100 protein) to detect recurrence is generally not recommended as low positive predictive values have been demonstrated. Ongoing research focuses on liquid biopsies, namely the detection of molecular alterations in plasma and serum of melanoma patients by characterization of circulating tumor cells and cell-free circulating tumor DNA [34,35]. This may provide valuable information on prognostic outcomes and assessment of treatment response or resistance in the future.

The National Comprehensive Cancer Network (NCCN), an alliance of 31 cancer centers in the United States, has released follow up recommendations per melanoma stage [1]. According to them, no routine imaging is recommended for stage 0 (in situ) melanoma. For patients with stage IA to IIA with no evidence of disease, routine imaging to screen for asymptomatic recurrence or metastatic disease is not recommended. Clinical visits should be scheduled every 6 to 12 months for 5 years and annually thereafter, as clinically indicated. Clinical examination in these visits should emphasize on the regional nodes and skin. For patients with stage IIB to IV (with no evidence of disease), scheduled visits should be conducted every 3 to 6 months for the first 2 years, every 3 to 12 months for the next 3 years and annually thereafter, as clinically indicated again emphasizing on the regional nodes and skin. Moreover, in these stages, imaging (chest x-ray, CT and/or PET/CT) every 3 to 12 months could be considered to screen for asymptomatic recurrence. Regarding central nervous system (CNS), a periodic brain MRI should be performed for up to 3 years to screen for asymptomatic brain metastases in high-risk patients with stage IIIC or higher melanoma, while more frequent surveillance is recommended for patients with prior brain metastases. However, routine imaging is not recommended after 3 to 5 years. Nonetheless, in any case and at any time of follow-up period, when clinically indicated, an appropriate imaging should be offered to evaluate specific signs or symptoms.

Finally, if relapse occurs, imaging is recommended to assess the extent of the disease. In addition, when complete surgical resection of relapse is not feasible and active non-surgical treatment is initiated, clinical examination and/or imaging may be appropriate throughout treatment to assess treatment response.

In Europe, follow up schemes vary among countries, ranging in frequency from 2 to 4 times per year for 5–10 years, again with higher-intensity strategies in more advanced stages and during the first years. Current European consensus-based interdisciplinary guidelines for melanoma have proposed an example of follow-up schedule examinations based on stage and is shown in Table 6 [2].

Irrespectively of the selected follow-up scheme, an individualized approach taking into consideration patient’s risk factors, such as risk for recurrence, prior primary melanoma, family history of melanoma and atypical mole syndrome, is optimal. Moreover, patients’ education must be an integral part of the surveillance strategy and should include:

- Communication on what to expect from follow-up examinations and why it is important to be compliant with the regular follow-ups.
- Awareness that family members often have an increased melanoma risk.
- Guidance on how to perform regular self-examination of the skin and peripheral lymph nodes.
- Information regarding correct sun exposure behavior.

## Conclusion

In conclusion, although there is still no universally adopted follow-up strategy program to monitor melanoma patients, current recommendations, as described above, could serve as a guide for clinicians while future prospective studies are necessary to better standardize this follow-up protocols.

## Figures and Tables

**Table 1:** Clinical staging according to AJCC 8th edition [1].

	T	N	M
Stage 0	Tis	N0	M0
Stage IA	T1a	N0	M0
Stage IB	T1b	N0	M0
	T2a	N0	M0
Stage IIA	T2b	N0	M0
	T3a	N0	M0
Stge IIB	T3b	N0	M0
	T4a	N0	M0
Stage IIC	T4b	N0	M0
Stage III	Any t, Tis	≥ N1	M0
Stage IV	Any T	Any N	M1

**Table 2:** Pathological staging according to AJCC 8th edition [1].

	T	N	M
Stage 0	Tis	N0	M0
Stage IA	T1a	N0	M0
	T1b	N0	M0
Stage IB	T2a	N0	M0
Stage IIA	T2b	N0	M0
	T3a	N0	M0
Stage IIB	T3b	N0	M0
	T4a	N0	M0
Stage IIC	T4b	N0	M0
Stage IIIA	T1a/b, T2a	N1a, N2a	M0
Stage IIIB	T0	N1b, N1c	M0
	T1a/b, T2a	N1b/c, N2b	M0
	T2b, T3a	N1a/b/c, N2a/b	M0
Stage IIIC	T0	N2b/c, N3b/c	M0
	T1a/b, T2a/b,	T3a N2c, N3a/b/c	M0
	T3b, T4a	Any N ≥ N1	M0
	T4b	N1a/b/c, N2a/b/c	M0
Stage IIID	T4b	N3a/b/c	M0
Stage IV	Any T, Tis	Any N	M1

**Table 3:** Definition of T according to AJCC 8th edition [1].

Category	Thickness	Ulceration

TX: Primary tumor cannot be assessed	N/A	N/A

T0: No evidence of primary tumor	N/A	N/A

Tis (in situ)	N/A	N/A

T1	≤1 mm	
	
T1a	<0.8 mm	Without ulceration
	
T1b	<0.8	With ulceration
	0.8–1.0 mm	With or without ulceration

T2	>1.0–2.0 mm	
	
T2a	>1.0–2.0 mm	Without ulceration
	
T2b	>1.0–2.0 mm	With ulceration

T3	>2.0–4.0 mm	
	
T3a	>2.0–4.0 mm	Without ulceration
	
T3b	>2.0–4.0 mm	With ulceration

T4	>4.0 mm	
	
T4a	>4.0 mm	Without ulceration
	
T4b	>4.0 mm	With ulceration

**Table 4:** Definition of N according to AJCC 8th edition [1].

Category	Number of Tumor-Involved Regional Lymph Node	Presence of In-transit, Satellite, and/or Microsatellite Metastases
NX: Patients in whom the regional nodes cannot be assessed	N/A	No
N0: No regional metastases detected	N/A	No
N1	1 tumor-involved node or in-transit, satellite, and/or microsatellite metastases with no tumor-involved node	
N1a	1 clinically occult (ie, detected by SLN biopsy)	No
N1b	1 clinically detected	No
N1c	No regional lymph node disease	Yes
N2	2 or 3 tumor-involved nodes or in-transit, satellite, and/or microsatellite metastases with 1 tumor-involved node	
N2a	2 or 3 clinically occult (ie, detected by SLN biopsy)	No
N2b	2 or 3, at least 1 of which was clinically detected	No
N2c	1 clinically occult or clinically detected	Yes
N3	4 or more tumor-involved nodes or in-transit, satellite, and/or microsatellite metastases with 2 or more tumor-involved nodes, or any number of matted nodes without or with in-transit, satellite, and/or microsatellite metastases	
N3a	4 or more clinically occult (ie, detected by SLN biopsy)	No
N3b	4 or more, at least one of which was clinically detected, or presence of any number of matted nodes	No
N3c	2 or more clinically occult or clinically detected and/or presence of any number of matted nodes	Yes

**Table 5:** Definition of M according to AJCC 8th edition [1].

Category	Anatomic Site	LDH level
M0: No evidence of distant metastasis	N/A	N/A
M1	Evidence of distant metastases	See below
M1a	Distant metastasis to skin, soft tissue including muscle, and/or nonregional lymph node	Not recorded or unspecified
M1a(0)		Not elevated
M1a(1)		Elevated
M1b	Distant metastasis to lung with or without M1a sites of disease	Not recorded or unspecified
M1b(0)		Not elevated
M1b(1)		Elevated
M1c	Distant metastasis to non-CNS visceral sites with or without M1a or M1b sites of disease	Not recorded or unspecified
M1c(0)		Not elevated
M1c(1)		Elevated
M1d	Distant metastasis to CNS with or without M1a, M1b, or M1c sites of disease	Not recorded or unspecified
M1d(0)		Normal
M1d(1)		Elevated

**Table 6:** Example of follow up schedule examinations based on melanoma stage proposed by European consensus-based interdisciplinary guidelines [2].

Stage	Clinical-dermatological examination			Lymph node sonography		Laboratory examination: LDH, S-100		CT neck, thorax, abdominal, pelvic or PET/CT - MRI head	
Year	1 to 3	4 to 10	>10	1 to 3	4 to 10	1 to 3	4 to 10	1 to 3	4 to 10
IA	6 m	12 m	12 m	-	-	-	-	-	-
IB–IIB	3–6 m	6 m	12 m	6 m	-	-	-	-	-
IIC–IIIC	3 m	6 m	12 m	3–6 m	-	3–6 m	-	6 m	-
IIID	3 m	6 m	12 m	3–6 m	-	3–6 m	-	3–6 m	-
IV NED (resected, CR under therapy)	3 m	6 m	12 m	3–6 m	-	3–6 m	-	3 m	-
IV (M1a-M1d) (distant metastasis)	Individualized; otherwise staging every 12 weeks								

* NED= No evidence disease, CR= Complete response
